# Non-Linearity Analysis of Depth and Angular Indexes for Optimal Stereo SLAM

**DOI:** 10.3390/s100404159

**Published:** 2010-04-26

**Authors:** Luis M. Bergasa, Pablo F. Alcantarilla, David Schleicher

**Affiliations:** Department of Electronics, University of Alcalá, Alcalá de Henares, Madrid, Spain; E-Mails: pablo.alcantarilla@depeca.uah.es (P.F.A.); dsg68818@telefonica.net (D.S.)

**Keywords:** extended Kalman filter, localization, mapping, inverse depth parametrization, non-linearity analysis, 2D warping

## Abstract

In this article, we present a real-time 6DoF egomotion estimation system for indoor environments using a wide-angle stereo camera as the only sensor. The stereo camera is carried in hand by a person walking at normal walking speeds 3–5 km/h. We present the basis for a vision-based system that would assist the navigation of the visually impaired by either providing information about their current position and orientation or guiding them to their destination through different sensing modalities. Our sensor combines two different types of feature parametrization: inverse depth and 3D in order to provide orientation and depth information at the same time. Natural landmarks are extracted from the image and are stored as 3D or inverse depth points, depending on a depth threshold. This depth threshold is used for switching between both parametrizations and it is computed by means of a non-linearity analysis of the stereo sensor. Main steps of our system approach are presented as well as an analysis about the optimal way to calculate the depth threshold. At the moment each landmark is initialized, the normal of the patch surface is computed using the information of the stereo pair. In order to improve long-term tracking, a patch warping is done considering the normal vector information. Some experimental results under indoor environments and conclusions are presented.

## 1. Introduction

Real-time egomotion estimation has a key role in robotics and computer vision applications. Ever since the seminal work by Broida *et al.* in the early nineties [1, 2], egomotion estimation (also known in the robotics literature as Simultaneous Localization and Mapping, SLAM) has captured the attention of researchers and the interest of using cameras as sensors has grown considerably due to mainly three reasons: cameras are cheaper than commonly used scan-lasers, they provide rich visual information about scene elements and they are easy to adapt for wearable systems. According to this, the range of SLAM based applications has spread to atypical robotic environments such as non-invasive surgery [3], augmented reality [4] and vehicle localization [5].

In this work, a 6DoF metric Stereo SLAM with a hand-held camera as the only sensor, is proposed for people egomotion estimation in order to provide on-line metric maps and localization to the users. Our system lays down the bases towards a high level 6DoF SLAM for the visually impaired. Since the users of the system are humans, there are no special constraints about camera movement (*i.e.*, the camera does not need to look at one side), although camera motion is expected to be smooth and the user has to walk at normal walking speeds 3–5 km/h. The main advantages of using a stereo system instead of a monocular one are described in [6]. With a stereo camera, we can obtain directly by triangulation an estimate of a 3D point depth and its associated uncertainty, whereas for the monocular case other strategies have to be done such as calibration grids, particle filtering [7] or undelayed initialization of features [8]. In addition, the maps that are obtained with a single camera are up to scale, since scale is not observable for a monocular camera.

We think that vision-based localization and mapping systems can provide the visually impaired with information about their current position and orientation and/or guide them to their destination through diverse sensing modalities [9]. Moreover, vision systems can also provide scene understanding [10] allowing visually impaired users to have a more effective navigation through space.

Some interesting works about navigation assistance for the visually impaired using vision and other sensors such as GPS can be found in the literature [11, 12]. In [11] map-based prior for localization is proposed as an useful help for a system based on GPS to localize blind people in urban environments, whereas Saez *et al.* presented in [12] a 6DoF stereo visual SLAM for the visually impaired. In their work, egomotion estimation is done by a point matching algorithm integrating 3D and 2D information. Mapping is done through a randomized global entropy minimization algorithm, considering othogonal scenarios, with difficult extension to non-orthogonal environments. In addition, the last system does not fulfil real-time constraints.

Our system follows a Davison’s SLAM approach [7], *i.e.*, a few high quality features are tracked and used to compute the position of the camera creating a sparse map of high quality textured landmarks using an Extended Kalman Filter (EKF). Paz *et al.* proposed in [13] a 6DoF Stereo EKF-SLAM system with stereo in hand for large indoor and outdoor environments. The inverse depth parametrization proposed by Civera *et al.* [8] for the MonoSLAM approach is adapted to the StereoSLAM version so as to provide distance and orientation information. Point features are extracted from the images and are classified as 3D features if the disparity is enough, or stored as inverse depth features otherwise. Their Visual SLAM algorithm generates conditionally independent local maps and finally, the full map is obtained using the Conditionally Independent Divide and Conquer algorithm, which allows constant time operation most of the time [14]. Although results are good considering large maps in indoor/outdoor environments, the range of camera movements is limited, since no patch adaptation is done and only 2D image templates correlations are carried out in the matching process. By means of an empirical analysis, they suggest choosing a threshold of depth 5 m, for switching between inverse depth and 3D features.

We introduce an adaptation of well-known techniques in the Robotics community and apply them to the problem of people egomotion for assisting the visually impaired community in navigation purposes. The two main contributions of our work, are the determination of a depth threshold for switching between inverse depth and 3D features by means of a non-linearity analysis, and a new 2D homography warping method considering information from both cameras of the stereo pair. This article is organized as follows: The general structure of the system is explained in Section 2.. In Section 3. the non-linearity analysis of depth and angular information and how to obtain an optimal depth threshold for switching between 3D and inverse depth features are explained. Then, in Section 4. we briefly explain the details of our EKF SLAM implementation. In Section 5. the 2D homography warping for patch adaptation is explained. Finally, some experimental results are shown in Section 6. Conclusions and future works are presented in Section 7.

## 2. System Structure

**Figure 1.**
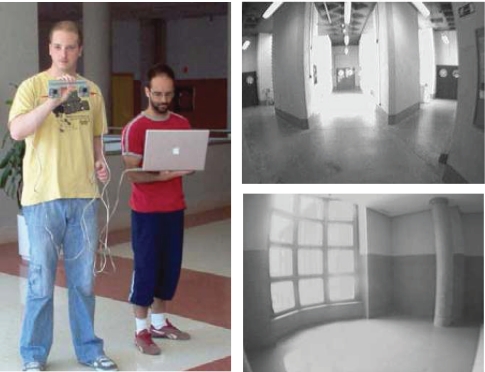
Stereo vision system for our 6DoF visual SLAM.

Our system consists of a hand-held stereo camera with wide angle lenses and a laptop for image processing. Figure 1 depicts our stereo system and the type of indoor environments where the experiments have been done.

The global state vector *X* incorporates the information for the left camera and for the features. The camera state *X_c_* is composed of its 3D position using cartesian coordinates, the camera orientation in terms of a quaternion, and linear and angular speeds, which are necessary for the impulse motion model used for modelling the camera movement.

(1) $$X_{c[13,1]}={\left(X_{\mathrm{cam}},\;q_{\mathrm{cam}},\;v_{\mathrm{cam}},\;\omega_{\mathrm{cam}}\right)}^{T}$$

For representing a rotation, it is enough to use a three components vector since a rotation matrix is defined by only 3 DoF. However, quaternions add an extra dimension since in this way it is easier to compose sequenced rotations. This 4*D*-vector defines a rotation angle *θ* around the unit vector *u* = (*u_x_ u_y_ u_z_*)*^t^* in the following way:

(2) $$q_{\mathrm{cam}}=\left(\begin{array}{c} q_{0}\\ q_{x}\\ q_{y}\\ q_{z} \end{array}\right)=\left(\begin{array}{c} \cos(\theta /2)\\ u_{x}\cdot \sin(\theta /2)\\ u_{y}\cdot \sin(\theta /2)\\ u_{z}\cdot \sin(\theta /2) \end{array}\right)$$

Two types of feature parametrization are used to provide orientation and depth information respectively. Depending on the depth of the feature as described in Section 3., features are initialized as inverse depth or 3D and are incorporated to the EKF SLAM algorithm. The final state vector *X* is shown in Equation 3:

(3) $$X={\left(X_{c},Y_{1\;3D}\cdots Y_{n\;3D},Y_{1\;\mathrm{INV}}\cdots Y_{m\;\mathrm{INV}}\right)}^{t}$$

Points of interest are extracted from the image using the Harris corner detector [15] and a subsequent subpixel refinement. When the camera moves, these features are tracked over the time to update the filter. In order to track a feature, image position is predicted in both cameras. Then, the feature appearance is transformed using a 2D homography according to Section 5., and a correlation search is performed inside a search area of high probability which is defined by the uncertainties of the feature and the camera. ZMCC (Zero Mean Cross Correlation) is used since its robustness against lighting changes. An intelligent feature management is implemented, so low-quality features are deleted from the state vector.

Due to the use of wide-angle lenses, it is necessary to use a distortion model correcting distorted images. Unlike other SLAM systems [6, 7] radial and tangential distortion are corrected using LUT (Look up tables), so images are corrected previous to processing. Two main advantages are obtained from using LUTs: firstly, this method is faster than working with the distorted images and then correcting the distorted projection coordinates, and secondly, the matching process is less critical if undistorted images are used.

### 2.1. 3D Features

For 3D features, the feature’s state vector encodes the information about the 3D position of the feature in the global map reference system.

(4) $$Y_{3D\;[3,1]}={\left(x,y,z\right)}^{t}$$

### 2.2. Inverse depth Features

**Figure 2.**
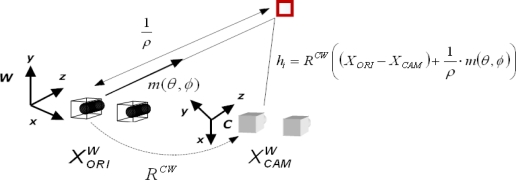
Inverse depth point coding.

For inverse depth features, the feature’s state vector encodes the information of the 3D optical center pose from which the feature was first seen *X_ori_*, the orientation of the ray passing through the image point (angles of azimuth *θ* and elevation *φ*) and the inverse of its depth, *ρ*. Figure 2 depicts the inverse depth point coding:

(5) $$Y_{\mathrm{INV}\;[6,1]}={\left(X_{\mathrm{ori}},\theta ,\phi ,\rho \right)}^{t}$$

In Figure 2, *m*(*θ*, *φ*) is the unitary ray directional vector from the camera to the feature. The angles of azimuth and elevation are defined as follows:

(6) $$\theta =\tan^{-1}\left(\frac{z}{x}\right)$$

(7) $$\phi =\tan^{-1}\left(\frac{\sqrt{x^{2}+z^{2}}}{y}\right)$$

## 3. Non-Linearity Analysis of Depth and Angular Information

Research in MonoSLAM has shown the benefits of using an inverse depth parametrization, since this parametrization allows undelayed initialization of features and mapping of features as infinity as well as close points [8, 16]. For the stereo case, the use or not of an inverse depth parametrization is not as critical as for the monocular case, since the depth can be determined. However, embracing an inverse depth parametrization approach is good for representing features at infinity providing bearing information and making the system more linear, which is better for the EKF. The main drawback of the inverse depth parametrization is that six values are needed for the parametrization instead of the only three values for a typical 3D parametrization, which produces a computational overhead in the EKF. Depending on the application, this computational overhead may be significant enough. The problem for the stereo case arises when it is necessary to decide at which depth the feature has to be parametrized as a 3D or as an inverse depth, this is, at which depth is better to use depth or angular information.

We propose to use a non-linearity analysis for finding an optimal depth threshold. A function is linear in an interval, if the first derivative is constant in that interval, and therefore, the second derivative is equal to zero. Considering the Taylor expansion for the first derivative of a continuous function *f* that depends of the variable *Z*:

(8) $$\frac{\partial f}{\partial Z}(z+\Delta z)\approx {\frac{\partial f}{\partial Z}|}_{z}+{\frac{\partial^{2}f}{\partial Z^{2}}|}_{z}\Delta z$$

Attending to the quotient between the second derivative and the first derivative, a dimensionless non-linearity index of the function *f* in relation with the variable *Z* can be found:

(9) $$L_{f}=\left|\frac{\frac{\partial^{2}f}{\partial Z^{2}}\cdot \Delta Z}{\frac{\partial f}{\partial Z}}\right|$$

Equation 8 can be expressed in terms of the non-linearity index *L_f_*:

(10) $$\frac{\partial f}{\partial Z}(z+\Delta z)\approx {\frac{\partial f}{\partial Z}|}_{z}\cdot (1+L_{f})$$

Observing Equation 10, two main conclusions are obtained:

1. If the non-linearity index *L_f_* is equal to zero for a point *Z_i_*, this implies that the function *f* is linear in interval Δ*Z*.
2. If the non-linearity index *L_f_* takes values higher than zero, this implies that the function *f* is not linear in the interval Δ*Z*.

### 3.1. Depth Non-Linearity

Considering an ideal stereo system, the depth of one point can be determined by means of the following equation:

(11) $$Z=\frac{f}{d_{x}}\cdot \frac{B}{u_{R}-u_{L}}=f_{x}\cdot \frac{B}{d_{u}}$$

where *f_x_* is the horizontal focal length in pixels, *d_u_* is the horizontal disparity in pixels and *B* is the baseline. The non-linearity index for the depth as a function of the horizontal disparity, is computed as follows:

(12) $$L_{Z}=\left|\frac{\frac{\partial^{2}Z}{\partial d_{u_{i}}^{2}}\cdot \Delta d_{u}}{\frac{\partial Z}{\partial d_{u_{i}}}}\right|$$

If the horizontal disparity *du* is isolated from Equation 11, we can express the depth non-linearity index as a function of the depth:

(13) $$L_{Z}=\frac{2\cdot \Delta d_{u}}{d_{u}}=\frac{2\cdot Z\cdot \Delta d_{u}}{f_{x}\cdot B}$$

### 3.2. Angular Non-Linearity

The angular non-linearity index *L_a_* is computed considering the angles of azimuth and elevation.

(14) La=Lθ+Lϕ =|∂2θi∂z2·Δz∂θi∂z|+|∂2ϕ i∂z2·Δz∂ϕ i∂z|

The expressions of the non-linearity index for the azimuth and elevation angle are respectively:

(15) $$L_{\phi }=\frac{x^{4}-2\cdot z^{4}+x^{2}(y^{2}-z^{2})}{z(x^{2}+z^{2})(x^{2}+y^{2}+z^{2})}\cdot \Delta z$$

(16) $$L_{\theta }=\frac{2\cdot z}{x^{2}(1+\frac{z^{2}}{x^{2}})}\cdot \Delta z$$

### 3.3. Optimal Depth Threshold

Given the baseline *B* of the stereo rig, the focal length in pixels *f_x_* and the image size (width (W), height (H)), we can estimate the stereo error from the maximum disparity *d_uMAX_* = *W* − 1 (minimum depth) to the minimum disparity *d_uMIN_* = 1 in incremental steps of 1 pixels as:

(17) $$\Delta Z_{i}=Z_{i}-Z_{i-1}=f_{x}\cdot B\left(\frac{1}{d_{u_{i}}-1}-\frac{1}{d_{u_{i}}}\right)=f_{x}\cdot B\cdot \frac{1}{d_{u_{i}}^{2}-d_{u_{i}}}$$

**Figure 3.**
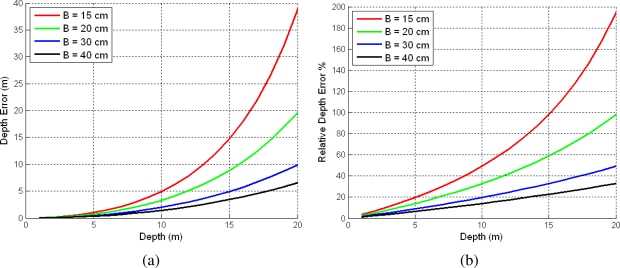
(a) Absolute and (b) relative depth estimation errors for a stereo rig considering a focal length *f_x_* = 202 pixels and image size 320 × 240, for different baselines.

Equation 17 shows the relationship between the depth accuracy and stereo rig parameters *f_x_*, *B* and image size (W,H). Figure 3 depicts the depth accuracy for different stereo baselines *B* considering fixed *f_x_* = 202 pixels and image size (*W* = 320, *H* = 240).

The type of graphs shown in Figure 3 have been proposed by Llorca *et al*. [29] to show the errors for depth estimates using a stereo rig. As it can be observed, depending on the baseline, the error in the depth estimate can be very high. In general, the higher the baseline the lower the error in the depth estimation. For example, for a typical wearable stereo rig device with a baseline of 15 cm, the error in depth if we try to estimate the 3D coordinates of a point located at a real distance of 10 m from the stereo rig, the relative error in depth Δ*Z* will be higher than 40% or more than 15 m in absolute terms. Adding those 3D points with very high uncertainties into the EKF can yield erroneous filter updates and propagation of errors. Therefore, it seems reasonable to find a depth threshold at which the accuracy of the angular measurements will be higher than depth measurements accuracy.

We performed several experiments in which we computed depth and angular non-linearity indexes for different baselines and considering the infinitesimal changes in disparity and depth as: Δ*u* = ±1 pixel and Δ*Z* = ±1 m. As can be seen in Equation 13, the depth non-linearity index depends on camera baseline and focal length. On the other hand, the angular non-linearity index (see Equation 14) depends only on relative 3D estimates perceived by each stereo rig. Therefore, even if we have different stereo rig configurations, the angular non-linearity index will be the same for each of the stereo rig settings, whereas the depth non-linearity index will be different for each configuration.

**Figure 4.**
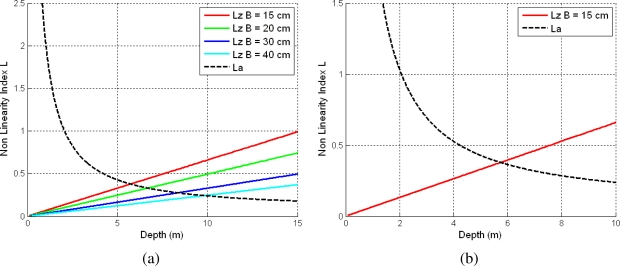
Depth and angular non-linearity indexes witch focal length *f_x_* = 202 pixels and image size 320 × 240. (a) Different stereo rig baselines (b) A zoomed version for our stereo rig configuration *B* = 15 cm.

Figure 4(a) depicts non-linearity indexes graphs considering different stereo rig baselines (B) and fixed focal length *f_x_* = 202 pixels and image size 320 × 240. Figure 4(b) depicts a zoomed version of the non-linearity graphs for our stereo rig configuration (*f_x_* = 202, *B* = 15 *cm, W* = 320, *H* = 240).

As it can be observed in Figure 4, both non-linearity indexes are equal at only one depth point. For depths higher than this threshold, the angular information is more linear than the depth one, and therefore an inverse parametrization is suitable in this case. On the contrary, for smaller depths a 3D parametrization is suitable. Table 1 shows the optimal depth threshold values for each of the considered baselines: For our stereo rig configuration, we suggest using a depth threshold *Z_t_* = 5.71 *m* as the optimal one for switching between both types of parametrizations. Our result is quite similar to the one obtained by Paz *et al.* in [13], where they found by means of an empirically analysis a threshold of 5 m considering a baseline of 12 cm.

## 4. EKF SLAM Overview

Assuming that Equation 3 denotes the state vector *X* and that its corresponding covariance matrix is denoted by *P*, the EKF implementation is described as follows, considering *k* as the step index:

1. **Prediction Step**
  (18) $$\hat{X}(k+1|k)=f(X(k|k))=f(k|k)$$
  (19) $$\hat{P}(k+1|k)=\frac{\partial f}{\partial X}(k|k)\cdot P(k|k)\cdot {\left(\frac{\partial f}{\partial X}(k|k)\right)}^{t}+Q(k)$$
2. **Update Step**
  (20) $$\hat{X}(k+1|k+1)=\hat{X}(k+1|k)+W(k+1)\cdot \eta {\left(k+1\right)}_{\mathrm{tot}}$$
  (21) $$P(k+1|k+1)=P(k+1|k)-W(k+1)\cdot S(k+1)\cdot {\left(W(k+1)\right)}^{t}$$

where *Q* and *S* are respectively the *process noise* and *measurement uncertainty* covariances. In addition, *η_tot_* is the *innovation* vector, *i.e.*, it means the difference between the current measurement vector and the predicted measurement one: (*η_tot_* = *z_tot_* − *h_tot_*).

### 4.1. Motion Model

For modeling the camera motion between two consecutive frames, we use a general motion model to predict the camera pose in the next frame. Since in this case we are using a hand-held camera, we assume 6DoF and we expect smooth motions. Our motion model assumes that the camera linear and angular velocities may change in every frame, but they are expected to be constant in average. This means, that the camera movements are approximated using the linear and angular velocity motion model [7]. This model assumes that in each time step the unknown linear (*a⃗^W^*) and angular accelerations (*α⃗^C^*) cause impulses of linear (*V⃗^W^*) and angular (Ω⃗*^W^*) velocities. According to this, the noise vector *n⃗* can be expressed as:

(22) $$\vec{n}=\left(\begin{array}{c} {\vec{V}}^{W}\\ {\vec{\Omega }}^{C} \end{array}\right)=\left(\begin{array}{c} {\vec{a}}^{W}\cdot \Delta t\\ {\vec{\alpha }}^{C}\cdot \Delta t \end{array}\right)$$

where *W* and *C* denote transformations with respect the world and left camera coordinate frame respectively. In order to predict the next state of the camera the function *f_v_* is defined:

(23) $$f_{v}=(X_{\mathrm{cam}}+V_{\mathrm{cam}}\cdot \Delta t,q_{\mathrm{cam}}\times q(\omega \cdot \Delta t),v_{\mathrm{cam}},\omega_{\mathrm{cam}})$$

The function *q* (*ω* · Δ*t*) represents the transformation of a 3 components vector into a quaternion. Assuming that the map does not change during the whole process, the absolute feature positions *Y_i_* are the same from one step to the next one. Assuming that linear and angular speeds are independent, the covariance matrix of the noise vector *n⃗* will be diagonal. Then, the process noise covariance *Q* can be computed via the corresponding Jacobian function as follows:

(24) $$Q=\frac{\partial f_{v}}{\partial \vec{n}}\cdot P_{\vec{n}}\cdot {\left(\frac{\partial f_{v}}{\partial \vec{n}}\right)}^{t}$$

### 4.2. Measurement Model

Visual measurements are obtained from the set of map points that are *visible* for a given camera pose. In our system, the measurement prediction vector for each feature is composed of the image projections of that 3D point in both left and right cameras with respect to the current camera pose, *i.e.*, *h_i_* = (*u_L_ v_L_ u_R_ v_R_*)*^t^*.

In order to decide, which features are going to be measured, we predict the visibility of every feature in the map. In this way, we can predict if the appearance of a given feature is close enough to the original appearance when the feature was initialized. Our visibility criteria is based in a length and angle heuristic. Feature visibility is calculated considering the difference between the viewpoint from which the feature was initially seen and a new viewpoint. This difference in viewpoint has to be below some length and angle ratio, and predicted to lie within the image, in order to predict the feature as visible. Usually the feature is expected to be visible if the length ratio $\left|h_{i}^{3D}|/|h_{\mathrm{orig}}^{3D}\right|$ is close enough to 1 (in practice between 5/7 and 7/5) and the angle difference $\beta ={\text{cos}}^{-1}((h_{i}^{3D}\cdot h_{\mathrm{orig}}^{3D})/(|h_{i}^{3D}||h_{\mathrm{orig}}^{3D}|))$ is close to 0 (less than 45° in magnitude).

#### 4.2.1. Measurement Prediction

Prior to perform the actual measurement, we need to obtain the value of the predicted vector *h_i_* for each of the visible features. This vector can be obtained as the result of a coordinate frame change (from the world coordinate frame *W* to the camera coordinate frame *C*) and then projecting the resulting 3*D* vector into the image plane according to the camera calibration matrix *K* and stereo-rig calibration parameters.

(25) $$h_{i}=K\cdot R^{CW}(Y_{i}^{W}-X_{\mathrm{cam}}^{W})$$

#### 4.2.2. Measurement Search

In order to obtain the measurement vector for each feature *z_i_* we have to define a search area around the predicted projections to limit the search to a high probability area of finding a good measurement inside. This area is computed based on the uncertainty of the features 3*D* position, which is called *innovation covariance S_i_*. This covariance essentially depends on three parameters: The camera state uncertainty *P_XX_*, the feature position uncertainty *P_YY_* and the measurement noise *R_i_*. The expression for this covariance is obtained as follows:

(26) $$S_{i}=\frac{\partial h_{i}}{\partial X_{v}}\cdot P_{XX}\cdot {\left(\frac{\partial h_{i}}{\partial X_{c}}\right)}^{t}+\frac{\partial h_{i}}{\partial X_{c}}\cdot P_{XY_{i}}\cdot {\left(\frac{\partial h_{i}}{\partial Y_{i}}\right)}^{t}+\frac{\partial h_{i}}{\partial Y_{i}}\cdot P_{Y_{i}X}\cdot {\left(\frac{\partial h_{i}}{\partial X_{v}}\right)}^{t}+\frac{\partial h_{i}}{\partial Y_{i}}\cdot P_{Y_{i}Y_{i}}\cdot {\left(\frac{\partial h_{i}}{\partial Y_{i}}\right)}^{t}+R_{i}$$

As we have two different views, *S_i_* needs to be transformed into the projection covariances for both left and right views, *S_iL_* and *S_iR_* respectively. These two covariances can be obtained easily from the *S_i_* matrix. These two covariances define both elliptical search regions, which are obtained taking into account a certain number of standard deviations (usually 3) from the 2D Gaussians.

Once the search areas are defined, we try to measure each of the features. At the initialization stage of each feature we store an 11 × 11 2D image template centered on the interest point and also an estimate of its normal vector, assuming that the feature is located onto a plane. Then, we modify the original 2D image template according to the current camera pose with a 2D image warping that is described in Section 5. Then, we perform a correlation search over the whole search area and compare the best correlation value to a threshold value. Then, if the correlations in the two views (left, right) are good enough, the new measured projection coordinates are saved in order to perform the filter update. Otherwise, the feature is marked as *unsuccessfully measured*.

#### 4.2.3. Filter Update

To perform the filter update, the Kalman gain *W* must be obtained by means of the following expression:

(27) $$W=P\cdot {\left(\frac{\partial h}{\partial X}\right)}_{\mathrm{tot}}^{t}\cdot S^{-1}$$

For each individual feature, the Jacobians *∂h_i_*/*∂X_cam_* and *∂h_i_*/*∂Y_i_* are obtained from Equation 25, which conveniently grouped form the total Jacobian (*∂h*/*∂X*)*_tot_*. Following the same procedure, the vector *z_tot_* that contains all the measurements is formed as well.

### 4.3. Feature Management

In order to build the map incrementally, we need to define a criteria for adding new features and deleting those ones whose tracking was poor during previous frames. When a new feature is added into the system, the feature is initialized with its respective 3D feature uncertainty plus the current camera pose uncertainty. In the next steps, the rules for adding new features will be to maintain, at least, 10 visible features at the same time. In addition to that, there will have to be, at least, 7 successfully measured features at the same time in order to avoid the complete loss of the camera tracking.

Besides, some of the features that are in the total state vector can be *bad* features; *i.e.*, features that are often poorly textured and the ratio of successful measurement is low. This could be as a consequence of reflections, occlusions, *etc.* We delete any feature that has been unsuccessfully measured more than a half of the attempts. When a new feature is added to the filter, not only the total state vector *X* has to be modified, but also the total covariance matrix *P*. This is done by simply adding an extra row and column in *P*. In order to remove a certain feature, the total covariance matrix *P* will be modified by removing the corresponding row and column.

### 4.4. Switching between Inverse Depth and 3D Features

Harris corners are extracted from the images and are classified as 3D features or stored as inverse depth features, depending on the estimated optimal depth threshold. Once the features are predicted in the EKF prediction step, it is necessary to determine if the original parametrization of the features has to be changed (*i.e.*, if an inverse depth feature is now below the depth threshold and should adapt a 3D parametrization or viceversa). Besides, a constraint is imposed: the feature has to remain at least *m* frames (typically 15 frames) in its new parametrization state before the switching. This is done in order to avoid unnecessary switchings in case that the depth estimate is above and below the threshold in consecutive frames.

When an inverse depth feature is switched to a 3D parametrization, it is necessary to adapt the feature’s state and the covariances implied in the filtering process by means of Equation 28 for the feature’s state and Equations 29 and 30 for the covariances. In the same way we can easily switch between 3D features to inverse depth.

(28) $$Y_{3D\;[3,1]}=X_{ORI}+\frac{1}{\rho }\cdot m(\theta ,\phi )$$

(29) $$P_{{YY}_{3D\;[3,3]}}=\left(\frac{\partial Y_{3D}}{\partial Y_{INV}}\right)\cdot P_{{YY}_{INV}}\cdot {\left(\frac{\partial Y_{3D}}{\partial Y_{INV}}\right)}^{t}$$

(30) $$P_{{XY}_{3D\;[13,3]}}=P_{{XY}_{INV}}\cdot {\left(\frac{\partial Y_{INV}}{\partial Y_{3D}}\right)}^{t}$$

## 5. 2D Homography Warping

When a feature is going to be measured, the estimation of the left camera position and orientation, which are obtained both from the SLAM state vector, and the normal surface patch vector are used for transforming the initial image template appearance (due to changes in viewpoint) by warping the initial template using a 2D homography. Our approach is related to the previous works of [17, 18].

Considering two camera centered coordinate systems, the transformation between two generic coordinate systems *X*_1_ and *X*_2_ is defined by:

(31) $$X_{2}=R\cdot X_{1}+T$$

where R and T are the rotation matrix and the translation vector encoding the relative position of the two coordinate systems. If *X*_1_ is a point on the plane defined by Equation 32:

(32) $$\pi :a\cdot x_{1}+b\cdot y_{1}+c\cdot z_{1}+1=0$$

This is a plane which does not pass through the origin, and *n* = (*a, b, c*)*^t^* is the plane normal. According to this, the following relationship can be found:

(33) $$n^{t}\cdot X_{1}=-1$$

Using the previous equation, Equation 31 can be expressed as follows:

(34) $$X_{2}=R\cdot X_{1}-T\cdot n^{t}\cdot X_{1}=(R-T\cdot n^{t})\cdot X_{1}$$

And therefore, image positions in the two camera frames are related by the 2D homography:

(35) $$U_{2}=C_{2}\cdot (R-T\cdot n^{t})\cdot C_{1}^{-1}\cdot U_{1}$$

**Figure 5.**
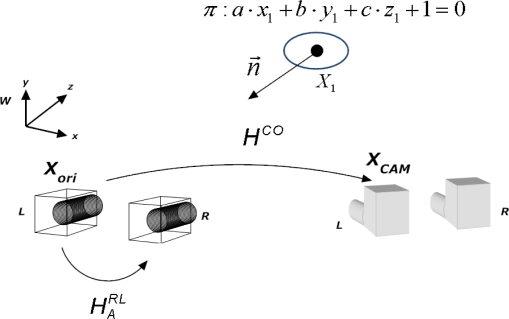
Stereo geometry and locally planar surfaces.

Figure 5 depicts the stereo geometry, and also the problems of obtaining the plane normal vector and the 2D homography for warping the initial image template using information from both cameras.

Equation 36 denotes the relationship between the left camera and the right camera coordinate systems:

(36) $$U_{R}=C_{R}\cdot (R^{RL}-T^{RL}\cdot n^{t})\cdot C_{1}^{-1}\cdot U_{L}$$

The previous equation depends on the rotation matrix *R^RL^* and the translation vector *T^RL^* between both cameras. The values of these matrices are known accurately, since they are estimated in a previous stereo calibration process. Supposing an affine transformation between left and right image patches, the affine transformation $H_{A}^{RL}$ can be expressed as:

(37) $$H_{A}^{RL}=C_{R}\cdot (R^{RL}-T^{RL}\cdot n^{t})\cdot C_{L}^{-1}$$

Both rotation and translation fall into the family of affine transformations [19]. This affine transformation can be computed easily by means of 3 correspondences of non-collinear points and with the assumption of locally planar patches. As it can be observed, Equation 37 depends on the plane normal vector *n*. From Equation 37 the product *T^RL^* · *n^t^* can be isolated. Denoting this product as *X*, it can be obtained as follows:

(38) $$X=T^{RL}\cdot n^{t}=R^{RL}-C_{R}^{-1}\cdot H_{A}^{RL}\cdot C_{L}$$

All the parameters of Equation 38 are known, since the affine transformation $H_{A}^{RL}$ has been previously computed, and the rest of implied matrices are known from the stereo calibration process. According to this, a system of 9 equations and 3 unknowns, which are the components of the plane normal vector, can be found:

(39) $$\{\begin{array}{ccc} n_{x}=\frac{X_{11}}{T_{x}} & n_{x}=\frac{X_{21}}{T_{y}} & n_{x}=\frac{X_{31}}{T_{z}}\\ n_{y}=\frac{X_{12}}{T_{x}} & n_{y}=\frac{X_{22}}{T_{y}} & n_{y}=\frac{X_{32}}{T_{z}}\\ n_{z}=\frac{X_{13}}{T_{x}} & n_{z}=\frac{X_{23}}{T_{y}} & n_{z}=\frac{X_{33}}{T_{z}} \end{array}$$

At the moment of a feature initialization, the plane normal vector is computed in the way it has been explained. Once this normal vector is estimated, the 2D homography between two different viewpoints can be determined using the estimation of the current left camera position and orientation and the left camera position and orientation: from the feature initialization viewpoint:

(40) $$U_{CAM}=C_{L}\cdot (R^{CO}-T^{CO}\cdot n^{t})\cdot C_{L}^{-1}\cdot U_{ORI}$$

where *R^CO^* and *T^CO^* are the rotation and translation matrices between the current left camera position and the reference position when the feature was initialized.

## 6. Experiments in Indoor Environments

In order to test the system performance, lots of indoor sequences have been tested. In this work, we present only the results of three of them. The cameras used were the Unibrain Fire-i IEEE1394 modules with additional wide-angle lens of 1.9 mm which provides a field of view of around 100° horizontal and vertical. Camera calibration is done in a previous setup process according to the one described in [20]. The camera provides a baseline of 15 cm, image resolution was 320 × 240 pixels and the images were B&W sequences. The acquisition frame rate was 30 frames per second. The sequences were processed on a laptop with an Intel Core 2 Duo processor at 2.4GHz. Our Visual SLAM algorithm is implemented in C/C++ and works in real-time (30 fps) under small environments whose number of landmarks is below 100 approximately. In our experiments, ground truth was obtained by means of a wheel odometer which measures the total trajectory length accurately in the horizontal and longitudinal axis X and Z respectively.

**Figure 6.**
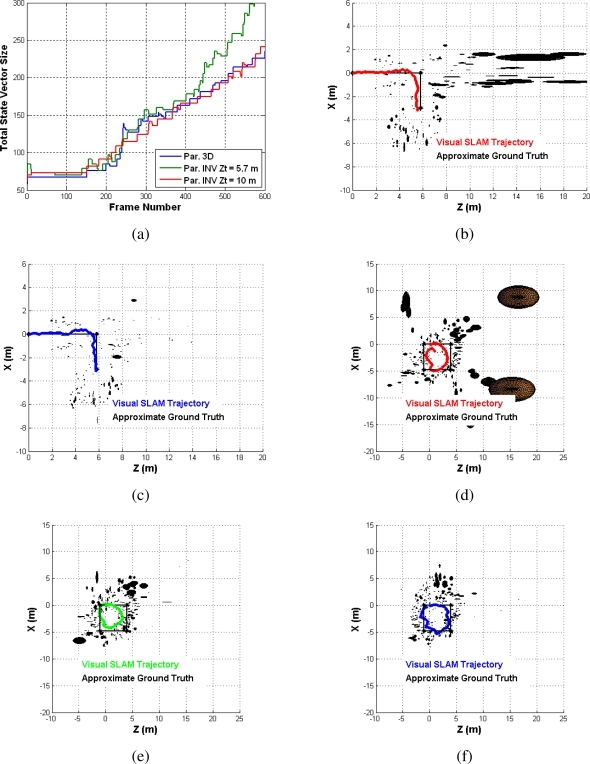
Inverse depth and 3D comparison. (a) Total state vector size, (b) Without inverse depth par. L sequence, (c) with inverse depth par. Z = 5.7 m L sequence, (d) without inverse depth par. loop sequence, (e) with inverse depth par. Z = 10 m loop sequence, (f) with inverse depth par. Z = 5.7 m loop sequence.

The first sequence is a typical corridor indoor sequence. The corridor has a length of 10 m, and the camera moves in a straight tilted left trajectory. This scenario is suitable for inverse depth parametrization, since we can find very far features that are parametrized as inverse depth points. We performed a comparison between inverse depth and 3D parametrization and studied three different cases: without inverse depth parametrization, with both parametrizations using two different depth thresholds of *Z_t_* = 10 *m* and *Z_t_* = 5.7 *m*. The second sequence was a typical L sequence of dimensions 3 m through the *X* axis and 6 m through the *Z* axis. Finally, the last sequence was a loop of dimensions 4.8 m through the *X* axis and 5 m through the *Z* axis. Figure 6(a) depicts the evolution of the state vector size for some frames of the L sequence. As it can be observed, the size of the state vector considering an inverse depth parametrization with a threshold of 5.7 m is bigger than in the rest of the cases, due to the computational overhead of using an inverse depth parametrization.

The final map and trajectory of the L and loop sequences are displayed in Figure 6, considering the different experiments. Table 2 shows the results of the comparison between the different analyzed cases. The legend of the parameters for this table is as follows:

- % **Inverse Features**: Is the percentage of the total number of features in the map that were initialized with an inverse depth parametrization.
- *ɛ_i_*: Is the absolute mean error in *m*, for the cartesian coordinates (*X*, *Z*).
- **Mean** *P_YY_* **Trace**: Is the mean trace of the covariance matrix *P_YY_* for each of the features that compose the final map. This parameter is indicative of the uncertainty of the features, *i.e.*, the quality of the map.

When a feature is predicted as visible and is going to be measured, we perform a correlation search over a high probability area of finding the measurement. For performing this correlation search, we can use the original image patch that was captured when the feature was initialized or we can modify the appearance of this original patch with respect to the current camera pose by means of the 2*D* warping explained in Section 5. In Table 3, we show a comparison between performing correlation with the original image patch and the transformed one by means of the 2*D* warping. The legend of the parameters of this table is as follows:

- **Case**: No patch transformation or 2*D* patch warping.
- **Sequence**: The test sequences for which we performed the comparison. We selected the corridor and L sequence. In the corridor sequence the changes in appearance are mainly due to changes in scale, whereas in the L sequence changes in appearance are mainly due to changes in scale and viewpoint.
- # **Features Map**: Is the total number of features in the map at the end of the sequence.
- # **Total Attempts**: Is the total number of feature measurement attempts during all the sequence.
- # **Successful Attempts**: Is the total number of successful feature measurement attempts during all the sequence.
- **Ratio**: Is the ratio between the number of successful measurement attempts and the total number of attempts.

According to Table 3, we can observe that by means of the proposed 2*D* warping we can increase the mean track length of a feature considerably with respect to the case of using the original patch and maximize the ratio between the successful measurement attempts and the total number of measurement attempts.

Respect to the processing time, real-time implementation imposes a time restriction, which shall not exceed 33 ms for a 30 frames/second capturing rate. Table 4 shows the information about processing time, considering inverse depth parametrization with a depth threshold of *Z_t_* = 5.7 m. The results were taken using a 2.0 GHz speed CPU. It can be observed that the most consuming steps are: Feature initialization, measurement and update. According to Table 4, the initialization of 15 features takes approximately 15 ms, since we have to run Harris corner detector, find the correspondence of interesting points on the left image and the right image by means of the epipolar search, obtain the 3D coordinates of the point and compute the values of the normal plane for the 2D warping. However, we have only to perform such an exhaustive initialization at the first frame, then we track features and only initialize new features when the number of visible features is small than a lower bound. In our system we also have an upper bound relative to the number of features that are visible for a given camera pose, and this bound is set to 15 for computational purposes. We only try to measure those features which are predicted to be visible. EKF filter update dominates processing time, since as long as we add new more landmarks to the filter the cost of the update is *O*(*n*^3^), being *n* the number of landmarks. For this reason, our system works only under real-time constraints under small environments whose number of landmarks is below 100 approximately. For mapping larger environments submapping strategies [21] or more efficient filtering methods such as [22] can be used.

## 7. Conclusions and Future Works

In this article we have presented a system that allows self-locating a stereo camera by combining depth and angular information from different natural landmarks. We think that our vision-based localization system can help in the future the visually impaired community assisting them in navigation purposes by either providing information about their current position and orientation or guiding them to their destination through diverse sensing modalities. One of the contributions of our work is the determination of an depth threshold for switching between inverse depth and 3D features by means of a non-linearity index analysis of the stereo sensor. In addition, the benefits of using an inverse depth parametrization for mapping features at infinity have been shown. However, depending on the application (the scenario and computation time constraints) the overhead due to the use of the inverse depth parametrization can be unnecessary, and higher values of depth for switching can be chosen, if the map quality is not altered. According to the results of Table 2, the simulation with the optimal threshold was the one that obtained better results in terms of absolute errors and features uncertainties in the final map. Furthermore, in the loop sequence, the loop is not closed correctly when only 3D parametrization is considered.

However, using the proposed depth threshold can exceed real-time constraints due to the inverse depth parametrization overhead. We are very interested in studying the use of a dynamic threshold as a function of the kind of environment, instead of the static one that is currently used, so as to obtain the same map quality keeping real-time constraints.

Considering 2D image templates and the normal vector of the plane that contains the point in the space improves the tracking considerably and it is better than using just 2D image templates. However, since the normal vector is only estimated once per feature, an update of the patch normals estimation would likely be of benefit. Moreover, we are interested in using scale invariant features and descriptors such as center surround extrema features [23] due to its suitability and good performance for Visual SLAM applications.

In further works, a high level SLAM will be developed for mapping outdoor large environments. We plan to do a similar submapping approach as the one described in [24]. We will obtain local maps of small size satisfying real-time demands (typically no more than 100 landmarks) by means of the described EKF-SLAM approach, then we will identify topologically each of the local maps by means of a series SIFT descriptors [25] for different key frames, and develop efficient algorithms for loop closure detection [26, 27]. Once we have detected a loop closure situation, we will perform structure from motion optimization by means of techniques such as bundle adjustment [28].

In addition, we are interested in fusing the stereo system with inertial sensors such as pedometers and/or GPS for outdoor experiments. Besides, the motion model must be improved, due to the great variability of movements that a person walking can do. Other interesting alternative can be using fast 6DoF visual odometry priors, replacing general camera motion models.

As we are interested in the application of Visual SLAM techniques for the visually impaired navigation, we plan getting some feedback from some visually impaired organizations.

## Figures and Tables

**Table 1.** Optimal depth thresholds for different stereo baselines and fixed focal length *f_x_* = 202 and image size (320, 240).

**Stereo Baseline (cm)**	**Depth Threshold (m)**
15	5.71
20	6.69
30	8.35
40	9.81

**Table 2.** Inverse depth and 3D comparison: absolute errors in trajectory and map uncertainty.

**Sequence**	**Case**	**% Inverse Features**	*ɛ_X_*	*ɛ_Z_*	**Mean***P_YY_***Trace**
Corridor	Without Inverse Par.	0.00	0.9394	0.4217	0.1351
Corridor	With Inverse Par., *Z_t_* = 10 *m*	5.23	0.9259	0.4647	0.0275
Corridor	With Inverse Par., *Z_t_* = 5.7 *m*	24.32	0.7574	0.3777	0.0072
L	Without Inverse Par.	0.00	0.5047	0.3985	0.1852
L	With Inverse Par., *Z_t_* = 10 *m*	7.85	0.5523	0.1017	0.0245
L	With Inverse Par., *Z_t_* = 5.7 *m*	19.21	0.5534	0.2135	0.0078
Loop	Without Inverse Par.	0.00	0.4066	0.9801	0.2593
Loop	With Inverse Par., *Z_t_* = 10 *m*	5.27	0.3829	0.63030	0.0472
Loop	With Inverse Par., *Z_t_* = 5.7 *m*	12.36	0.2191	0.3778	0.0310

**Table 3.** Comparison of patch matching techniques: no patch transformation and 2*D* warping.

**Case**	**Sequence**	**# Features Map**	**# Total Attempts**	**# Successful Attempts**	**Ratio %**
No Patch Transformation	Corridor	85	6,283	5,612	89.32
2D Warping	Corridor	68	6,398	5,781	90.35
No Patch Transformation	L	116	11,627	8,922	76.73
2D Warping	L	105	10,297	9,119	88.71

**Table 4.** Processing times.

**Filter Step**	**Time ms**
Feature Initialization (15)	18.00
Prediction	0.47
Measurement	10
Update	4.96
